# Phosphorus Addition Reduces Seedling Growth and Survival for the Arbuscular Mycorrhizal Tree *Cinnamomum camphora* (Lauraceae) and Ectomycorrhizal Tree *Castanopsis sclerophylla* (Fagaceae) in Fragmented Forests in Eastern China

**DOI:** 10.3390/plants12162946

**Published:** 2023-08-15

**Authors:** Jinliang Liu, Mengsi Zhou, Xue Li, Tianxiang Li, Haoyue Jiang, Luping Zhao, Shuman Chen, Jingying Tian, Wenjuan Han

**Affiliations:** 1 College of Life and Environmental Science, Wenzhou University, Wenzhou 325035, China; jinliang.liu@foxmail.com (J.L.); mengs_z@163.com (M.Z.); lixue0113@126.com (X.L.); tianx_lee@163.com (T.L.); 20211231114@stu.wzu.edu.cn (H.J.); 20211231140@stu.wzu.edu.cn (L.Z.); 20211231103@stu.wzu.edu.cn (S.C.); 20211231127@stu.wzu.edu.cn (J.T.); 2 Zhejiang Provincial Key Laboratory for Water Environment and Marine Biological Resources Protection, Wenzhou University, Wenzhou 325035, China

**Keywords:** phosphorus limitation, habitat loss, mycorrhizal symbiosis, subtropical, community dynamics

## Abstract

Global changes in nutrient deposition rates and habitat fragmentation are likely to have profound effects on plant communities, particularly in the nutrient-limited systems of the tropics and subtropics. However, it remains unclear how increased phosphorus (P) supply affects seedling growth in P-deficient subtropical fragmented forests. To explore this, we applied P to 11 islands in a subtropical Chinese archipelago and examined the results in combination with a contemporary greenhouse experiment to test the influence of P addition on seedling growth and survival. We measured the growth (i.e., base area) and mortality rate of seedlings for one arbuscular mycorrhizal (AM) and one ectomycorrhizal (EcM) tree species separately and calculated their relative growth rate and mortality when compared with P addition and control treatment on each island. We also measured three functional traits and the biomass of seedlings in the greenhouse experiment. Results showed that P addition significantly increased the mortality of AM and EcM seedlings and reduced the growth rate of EcM seedlings. The relative growth rate of AM seedlings, but not EcM seedlings, significantly decreased as the island area decreased, suggesting that P addition could promote the relative growth rate of AM seedlings on larger islands. The greenhouse experiment showed that P addition could reduce the specific root length of AM and EcM seedlings and reduce the aboveground and total biomass of seedlings, indicating that P addition may affect the resource acquisition of seedlings, thereby affecting their survival and growth. Our study reveals the synergistic influence of habitat fragmentation and P deposition, which may affect the regeneration of forest communities and biodiversity maintenance in fragmented habitats.

## 1. Introduction

Understanding the factors that affect biodiversity in fragmented habitats (e.g., islands) has long concerned conservation biologists and community ecologists [1,2,3]. Half of the planet’s forests lie within 500 m of an edge with an area of less than 10 hectares (ha) [4]. It is necessary to understand the key factors limiting the regeneration of plants in fragmented forests to restore vegetation and conserve biodiversity [5,6,7]. For plant communities, the growth and survival of seedlings is the initial stage of community assembly [8]. The reduction in nutrient content and increase in light availability with the decrease in habitat area (e.g., edge effect [9]) likely impact the growth and survival of seedlings, thus affecting biodiversity [7]. Therefore, exploring the key factors affecting the growth and survival of seedlings in fragmented forests will help understand the maintenance of biodiversity and vegetation restoration in fragmented habitats.

Phosphorus (P) limitation or nitrogen and P co-limitation occurs in many terrestrial ecosystems around the world [10,11]. Soil P availability could affect plant growth and survival [12], species diversity [13], and net primary productivity [14]. Soil P is a particularly limited resource in the highly weathered soils of lowland tropical and subtropical forests [15,16,17,18]. Furthermore, as a natural consequence of habitat fragmentation, there is an increasing influence of edge effects such as weathering, temperature, and rainfall that may influence soil P nutrient content [9] and further affect the early regeneration of plant communities [19], while it has been found that the woody plant species richness is positively correlated with the total P in a subtropical archipelago [20]. It is well understood that anthropogenic atmospheric deposition can increase P supply in ecosystems [21,22]. Despite this, there are few experimental studies on how increased P supply affects the growth and mortality of seedlings in a fragmented subtropical landscape. 

The symbiosis between plants and mycorrhizal fungi is one of the critical strategies to improve nutrient absorption by plants [23,24]. Ectomycorrhizal (EcM) and arbuscular mycorrhizal (AM) fungi are two main plant mycorrhizal symbionts for woody plant species in forests [25]. EcM fungi and AM fungi could improve the P absorption of EcM and AM plants [26,27,28] and further affect plant species diversity [29,30]. P addition can cause changes in the species composition of AM fungi in tropical montane forests [31], and habitat fragmentation could also cause changes in the community composition of mycorrhizal fungi [32]. A global-scale analysis showed that EcM tree species have a 12% higher P absorption efficiency than AM tree species, especially for deciduous species, while EcM and AM trees exhibit similar nutrient absorption efficiency for evergreen species [33]. Jiang et al. [34] also found that AM and EcM seedlings respond differently to environmental factors. Studies indicate that the relationship between species diversity and habitat size differs for AM and EcM plants in fragmented habitats [35,36]. However, we still need clarification as to the combined effect of P addition and habitat size on the growth of AM and EcM seedlings in fragmented forests, particularly in nutrient-limited subtropics [37,38]. 

Here, we aimed to test the synergetic effect of island area and P addition on the survival and growth of seedlings for one AM and one EcM tree species on islands in Thousand Island Lake (TIL) in China. Although studies have found that soil total P is one of the main factors affecting the richness of woody plant species on islands [20], and species diversity of AM but not EcM plants decreases with island area loss due to environmental filtering on islands in the TIL [36], there is still a lack of direct testing for the effect of P addition on the growth and mortality of AM and EcM seedlings on islands. To that end, we conducted P addition experiments on islands (i.e., the in situ experiment) and in the greenhouse (i.e., the ex situ experiment) separately to answer the following questions: (1) How does P addition affect the growth and mortality of seedlings on islands? (2) Does island area (i.e., fragment size) affect the influence of P addition on seedling growth rate and mortality? To explore the possible mechanism of P addition on seedling growth and mortality, a greenhouse experiment is applied to test (3) how P addition affects leaf and root functional traits and the biomass of seedlings.

## 2. Results

### 2.1. Effect of Phosphorus Addition on the Growth and Mortality of Seedlings 

**Figure 1:**
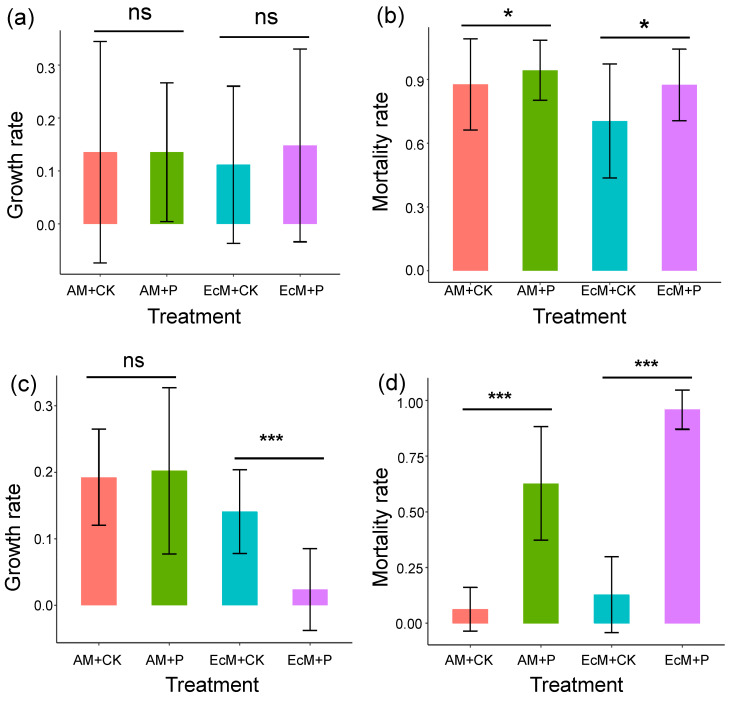
The difference in growth rate (**a**,**c**) and mortality rate (**b**,**d**) of *C. camphora* (AM) and *C. sclerophylla* (EcM) seedlings between phosphorus addition treatments (AM + P and EcM + P) and control treatments (AM + CK and EcM + CK) on the island experiment (**a**,**b**) and in the greenhouse experiment (**c**,**d**). The asterisk indicates significant differences between different treatments for *C. camphora* seedlings (AM + CK vs. AM + P) and *C. sclerophylla* seedlings (EcM + CK vs. EcM + P) using the ANCOVA method (* *p* < 0.05, *** *p* < 0.001, and ns: no significant).

In the P addition experiment on islands, the growth rate of *Cinnamomum camphora* (i.e., AM tree species) and *Castanopsis sclerophylla* (i.e., EcM tree species) seedlings showed no significant difference between the control treatment and the P-addition treatment (Figure 1a), while the P-addition treatment significantly increased the mortality rate of *C. camphora* (AM) and *C. sclerophylla* (EcM) seedlings when compared with the control treatment (Figure 1b). 

In the greenhouse experiment, the growth rate of *C. camphora* (AM) seedlings showed no significant difference between the control treatment and the P-addition treatment, but the growth rate of *C. sclerophylla* (EcM) seedlings was significantly lower in the P-addition treatment than the control treatment (Figure 1c). Similar to the results on islands, mortality was significantly higher under P-addition treatment than control treatment for both *C. camphora* and *C. sclerophylla* seedlings (Figure 1d).

### 2.2. Relationships between Island Area, Relative Growth Rate, and Relative Mortality Rate

**Figure 2:**
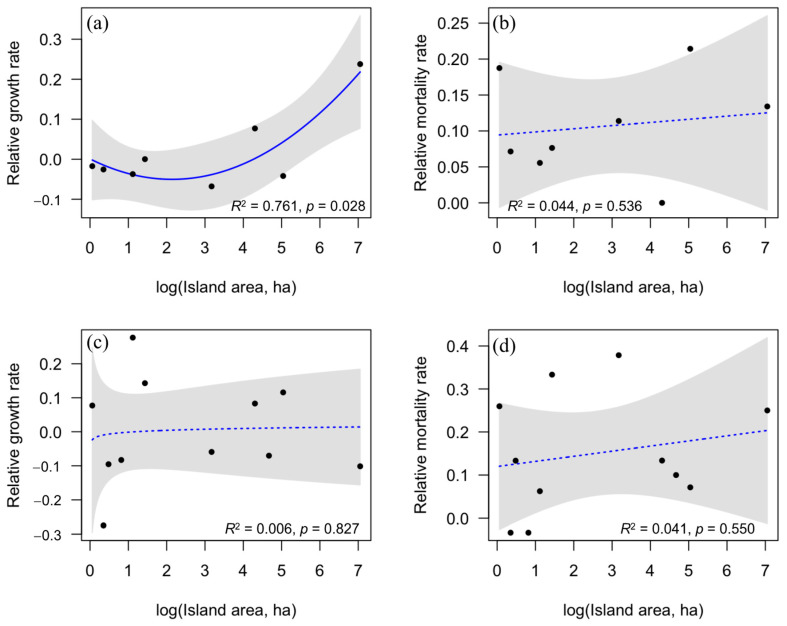
Relationships between island area and relative growth rate (**a**,**c**) and relative mortality rate (**b**,**d**) for *C. camphora* seedlings (**a**,**b**) and *C. sclerophylla* seedlings (**c**,**d**) on islands. Blue lines represent mode fit, and gray shaded areas represent 95% confidence intervals.

When the value of the difference in growth rate or mortality rate between the P-addition treatment and the control treatment (i.e., relative growth rate or relative mortality rate) was calculated on islands, we found a significant relationship between the relative growth rate of *C. camphora* seedlings and island area (Figure 2a, *R*^2^ = 0.761, *p* = 0.027), but not the relative mortality rate of *C. camphora* seedlings (Figure 2b). Neither the relative growth rate nor the relative mortality rate of *C. sclerophylla* seedlings showed any relation to island area (Figure 2c,d). 

### 2.3. Effects of Phosphorus Addition on Functional Traits 

**Figure 3:**
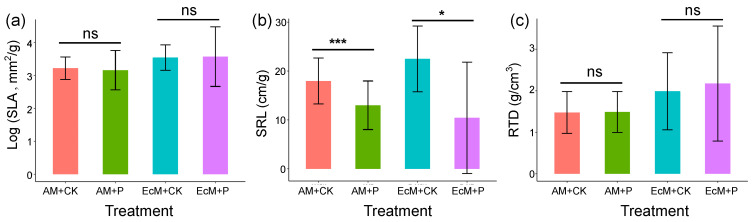
The difference in (**a**) log-transformed specific leaf area (SLA; mm^2^/g), (**b**) specific root length (SRL, cm/g), and (**c**) root tissue density (RTD, g/cm^3^) between phosphorus addition treatment (AM + P and EcM + P) and control treatment (AM + CK and EcM + CK) for *C. camphora* (AM) and *C. sclerophylla* (EcM) seedlings. The asterisk indicates significant differences between different treatments for *C. camphora* seedlings (AM + CK vs. AM + P) and *C. sclerophylla* seedlings (EcM + CK vs. EcM + P) (* *p* < 0.05, *** *p* < 0.001, and ns: no significant).

**Figure 4:**
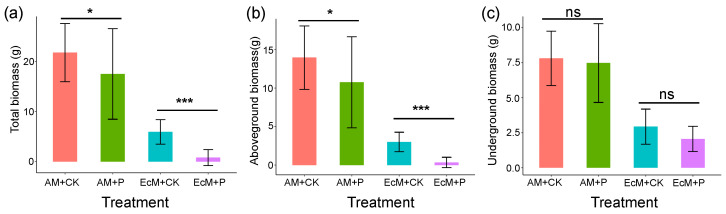
The difference in (**a**) total biomass, (**b**) aboveground biomass, and (**c**) underground biomass between phosphorus addition treatment (AM + P and EcM + P) and control treatment (AM + CK and EcM + CK) for *C. camphora* (AM) and *C. sclerophylla* (EcM) seedlings. The asterisk indicates a significant difference between different treatments for *C. camphora* seedlings (AM + CK vs. AM + P) and *C. sclerophylla* seedlings (EcM + CK vs. EcM + P) (* *p* < 0.05, *** *p* < 0.001, and ns: no significant).

There was no significant difference in specific leaf area (SLA) between the P-addition treatment and the control treatment for both *C. camphora* (AM) and *C. sclerophylla* (EcM) seedlings (Figure 3a). However, the specific root length (SRL) of *C. camphora* and *C. sclerophylla* seedlings was significantly higher in the P-addition treatment than the control treatment (Figure 4b). Interestingly, there was no significant difference in root tissue density (RTD) between the P-addition treatment and the control treatment for *C. camphora* and *C. sclerophylla* seedlings (Figure 3c).

The P-addition treatment significantly decreased the total biomass of *C. camphora* and *C. sclerophylla* seedlings compared with the control treatment (Figure 4a), and this reduction of total biomass after P addition was mainly caused by the reduction of aboveground biomass (Figure 4b) rather than underground biomass (Figure 4c).

## 3. Discussion

Global changes in nutrient deposition rates and habitat fragmentation are likely to profoundly affect plant communities, particularly in the nutrient-limited systems of the tropics and subtropics [38]. It may be accompanied by a reduction in soil nutrients and soil microbial activity associated with habitat loss [39]. Our study tested whether P deposition could improve the survival and growth of seedlings in subtropical fragmented forests known to be P-limited. Interestingly, we found that P addition could not improve the growth and survival of seedlings of *C. camphora* (i.e., AM tree species) and *C. sclerophylla* (i.e., EcM tree species). 

Many studies showed that P limits seedling growth in rainforests and subtropical forests, and P addition could enhance the survival of seedlings [30,40]. However, several studies have found the opposite results, which are consistent with our findings (Figure 1). For example, Newbery et al. [41] found that the EcM seedlings survived and grew better in the control treatment than in the P-addition treatment in tropical low-P soils, and Manu et al. [42] also found that the P addition was not pronounced in promoting tree growth and carbon accumulation rates in forests on highly weathered soils. Furthermore, some research suggests that long-term P addition could significantly decrease the survival rate of seedlings in a primary tropical forest [43]. These inconsistent results of seedling growth responses to P addition may depend on the mycorrhizal associations, studied species, nutrient level, and ecosystem types [43,44,45]. Together with our results, these results indicate that increased P supply alone may not generally increase tree growth and result in higher survivorship in ecosystems with low P availability, suggesting that other studies in relatively fertile soil may be limited [46]. In this study, we selected one species of AM tree (*C. camphora*) and one species of EcM tree (*C. sclerophylla*) for the P addition experiment. Both species were common in the study region. Although AM plants and EcM plants have different strategies for accessing phosphorus and some species of AM plants or EcM plants respond differently to P addition [30], we found that the two selected species demonstrated the same response to P addition. In our study, the mortality rates of *C. camphora* and *C. sclerophylla* seedlings increased when P was added in both the island and greenhouse experiments. These results further suggest that a mycorrhizal plant’s competitive advantage relative to a non-mycorrhizal species may be reduced in fragmented forests, probably due to a shift from commensalism to parasitism as increased nutrient availability reduces the positive influence of mycorrhizal plants. In addition, the phosphorus requirement differs between pioneer tree species and late-successional trees. Especially the selection on phosphorus acquisition and use may be strongest for pioneer species with high phosphorus demand [47]. While the two selected species, *C. camphora* and *C. sclerophylla,* were late-successional tree species in subtropical forests. This result may also be related to the species’ demand for phosphorus. 

When considering the synergistic effect of island area and P addition on the survival and growth of seedlings, we found that P addition has a more positive influence on the relative growth rate of *C. camphora* seedlings on larger islands. However, we did not identify any relationship between the island area and the relative growth rate of *C. sclerophylla* seedlings. Previous studies in the study system have found that total P was one limiting factor affecting species diversity, especially on smaller islands [20]. Li et al. [36] further found a significant positive correlation between the diversity of AM plants and island area, but not EcM plants. The decrease in island area and the commensurate loss in AM fungal diversity may decrease the extent to which AM fungi colonize plant roots and consequently reduce the nutrient absorption and growth of AM plants [48]. Therefore, *C. camphora*, as AM plants, may be more susceptible to P limitations on islands. In addition, as the island area decreases, other environmental factors, such as nitrogen (N) availability and soil thickness, will also limit plant growth [42,49]. Thus, only increasing P may not alleviate the impact of elements on plant growth restrictions on smaller islands but could promote plant growth on larger islands. However, the EcM tree species, such as *P. massoniana*, were the dominant tree species on all islands, and environmental factors did not limit the EcM tree diversity due to the EcM plant’s high nutrient absorption capacity [36]. Similarly, as an EcM plant species, this may be why P addition has no significant effect on the relative growth rate of *C. sclerophylla* seedlings on islands. 

To test the influence of P addition on leaf and root functional traits and biomass of seedlings in a controlled environment, we used a greenhouse experiment, which found that adding P can significantly decrease specific root length (Figure 3), total biomass, and aboveground biomass (Figure 4). Generally, species associated with rapid resource acquisition tend to have roots with higher specific root length and lower tissue density [50]. Our results indicate that P addition is likely to affect resource acquisition by reducing specific root length. For other species in the subtropical zone, e.g., *P. massoniana* (EcM plant) and *Schima superba* (AM plant), Liu et al. [51] found that P addition alone did not significantly affect seedling’s biomass and specific root length after one year, but N + P additions could significantly increase biomass and specific root length. In contrast, the study in subtropical forests found that P addition could significantly increase the biomass of seedlings after six months, such as *S. superba*, *Cinnamomum porrectum* (AM plant), *Castanopsis fissa* (EcM plant), and *Castanopsis faberi* (EcM plant) [30]. The differences between these findings are likely influenced by the studied species, the studied systems (e.g., mainland vs. island), and the time of phosphorus addition. In this study, we only selected one AM plant species and one EcM plant species to do the P addition experiment in a short time. EcM trees and AM trees both include late-successional and pioneer tree species [52], and some species of AM plants or EcM plants probably respond differently to P addition [30]. Considering the differences between fragmented and continuous habitats and different species having different demands for phosphorus, additional studies are crucial to advance our understanding of the mechanisms of multiple species communities in a long-term survey in fragmented forests.

Combining the results on islands and in the greenhouse, we suggest possible reasons for the increase in mortality and decrease in growth rate of *C. camphora* and *C. sclerophylla* seedlings when P was added on the studied islands. First, adding P may limit the resource acquisition of seedling roots on islands (represented by a decrease in specific root length), consequently reducing the seedling growth rate and increasing mortality. Second, P absorption by vascular land plants requires additional energy costs (i.e., carbon costs) [53]. As a result, increased carbon costs without enhanced aboveground carbon assimilation could potentially exhaust plant carbon storage [43], as we found decreases in total biomass and aboveground biomass in the P-addition treatment (Figure 4). Furthermore, increased P addition might weaken the associations between plants and mycorrhizal fungi, and seedlings are consequently more vulnerable to pathogens or other factors (such as allelopathy) [25]. 

## 4. Materials and Methods

### 4.1. Study Site

**Figure 5:**
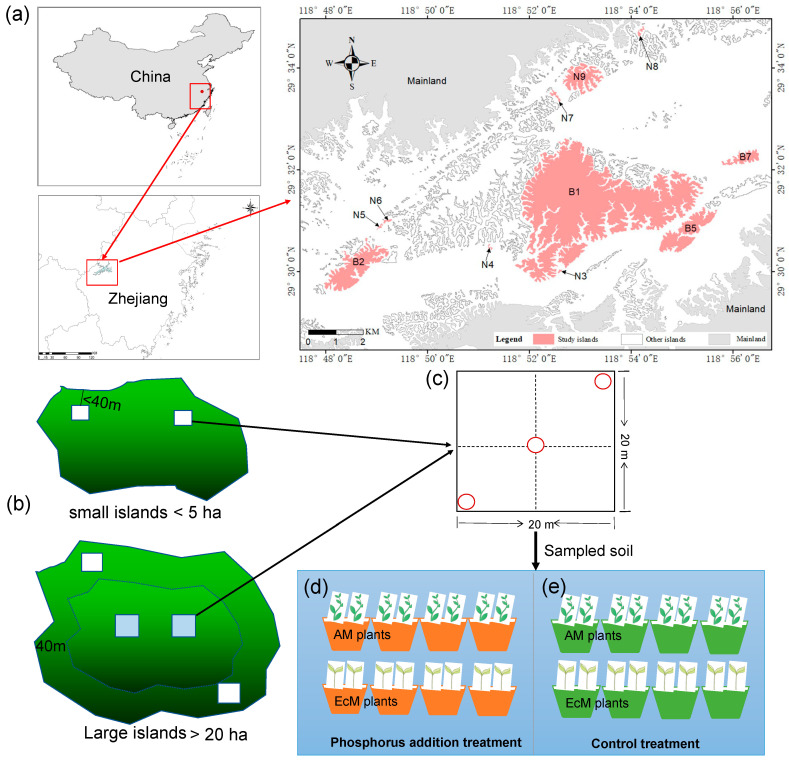
Experimental design. Eleven islands were selected for the Thousand Island Lake (TIL), which is located in eastern China (**a**). Two edge plots (20 m × 20 m, the distance to island edge less than 40 m) were set on small islands (<5 ha), and two edge plots and two interior pots (the distance to island edge larger than 40 m) were set on large islands (>5 ha) (**b**). Three subplots (red circle) were set along the diagonal in each plot, and a phosphorus addition experiment on *C. camphora* (AM plants) and *C. sclerophylla* (EcM plants) seedlings was applied in each subplot on islands (**c**). The soil samples from the three subplots in each plot were collected and mixed as a soil sample for a subsequent phosphorus addition treatment (**d**) and control treatment (**e**) experiment in greenhouses for *C. camphora* (AM plants) and *C. sclerophylla* (EcM plants) seedlings.

Our experiment was conducted at the Thousand Island Lake (TIL), Zhejiang Province, eastern China (29°22′–29°50′ N, 118°34′–119°15′ E) (Figure 5) [54]. The TIL was created by the inundation following dam construction on the Xin’an River in 1959. As a result, 1078 islands with an area above 0.25 ha were formed from the mountain tops. This region has been protected as a national park in China since 1962, and most of the islands have not experienced significant human disturbance. At present, the islands are dominated by *Pinus massoniana* in the canopy and broad-leaved plants in the sub-canopy and understory. Due to its clear formation history, uniform matrix, and obvious island boundary, this anthropogenic archipelago provides excellent opportunities to understand the assembly of plant communities in fragmented landscapes [20]. 

### 4.2. Selection of Experimental Islands and Sampling Sites

To test the effect of P addition on seedling growth on islands, we selected 11 islands with areas ranging from 1.06 to 1153.88 ha for our P-addition experiments (Figure 5a) [36,54]. Referring to the methods of Liu et al. [55] and Li et al. [36], we set up two edge plots (20 m × 20 m) within 40 m of the edge of the small island and added two interior plots above 40 m of the edge of the large island, depending on the area of the islands. Only edge plots were established on six small islands smaller than 5 ha because all plots were close to the island edge due to the limitation of island area, and two edge plots and two interior plots were established on five large islands with an area above 5 ha (Figure 5b). In total, 32 plots were established on all islands, reflecting 22 edge plots and 10 interior plots [36,54]. 

### 4.3. Phosphorus Addition Experiment on Islands

One species of ectomycorrhizal (EcM) tree (i.e., *C. sclerophylla*) and one species of arbuscular mycorrhizal (AM) tree (i.e., *C. camphora*) were selected in our experiment. These two tree species are commonly distributed in the study region and are often used for restoration. Seedlings of the two selected species were purchased from a local garden company and cultivated with seeds in the same year under the same growth conditions. 

To test the effects of island aera and P addition on the growth of seedlings of AM and EcM plants, we set up three subplots along the diagonal of each plot on each island (Figure 5c). In each subplot, a control treatment (CK) and a P-addition treatment were set up for AM seedlings and EcM seedlings separately: (a) control treatment (i.e., AM + CK, EcM + CK), and (b) P-addition treatment (i.e., AM + P, and EcM + P). In April 2022, three seedlings of each species were evenly planted in a 1 m × 1 m area for each treatment, so that a total of 12 seedlings were planted in each subplot. During the first two weeks after the seedlings were planted, we replaced the dead or poorly growing seedlings with new ones. In August 2022, we began the P-addition treatment. Based on the background P content in local soil and referring to previous studies on P deposition and nutrient limitation in tropical and subtropical forests [56], 10 g of P was added annually to each 1 m × 1 m area for P-addition treatment. P addition was carried out using 1 g of P (Na_3_PO_4_, trisodium phosphate) dissolved in 1.8 L of water and sprayed onto the soil surface using a sprayer. An equal amount of water (1.8 L) was added to the control area for control treatment. The P-addition treatment was repeated once a month for 5 months. The seedling height, base area, and mortality of seedlings were monitored monthly.

### 4.4. Phosphorus Addition Experiment in the Greenhouse

Soil samples were collected at 0–30 cm depth from three sampling points in each plot on islands (Figure 5). These soil samples were mixed and taken back to the greenhouse for our P addition experiment. The mixed soil taken from each plot on the islands was evenly filled into the 16 pots (specification: 23 cm × 18 cm × 21.5 cm) and thoroughly mixed with sand (v 1:1). The field soil and sand mixture could guarantee homogeneous soil nutrients among the 16 pots while somewhat retaining the soil properties of a specific plot on the island. In July 2021, for each AM and EcM plant species, we randomly selected and transplanted seedlings into eight pots (two seedlings per pot), with four pots for P-addition treatment and four pots for control treatment (Figure 5d,e). Two treatments were set up in the greenhouse experiment to reflect the island experiment. In total, 512 pots were set (32 plots × 2 species/plots × 8 pots/species = 512 pots). During the following two weeks after the transfer of seedlings into pots, we removed the seedlings that were dead or poorly growing due to injuries and replaced them with new seedlings. In August 2021, we began the P addition experiment to investigate the response of seedling growth to the P addition. Based on the background value of P content in the local soil, 0.24 mg P (Na_3_PO_4_, trisodium phosphate) was added to every 1 g of soil, and 10 mL of water was used to dissolve the phosphate so that it could be evenly distributed in the soil [30]. A control treatment received an equal volume of water. The P-addition treatments were repeated once every month for 10 months. We regularly watered the seedlings and monitored seedling heights every month.

### 4.5. Measurement of Functional Traits 

At the end of the experiment in the greenhouse in July 2022, all seedlings were harvested, washed to remove any attached soil, and separated into stem, leaf, and root for laboratory analysis. Three leaf and root functional trait variables were recorded: specific leaf area (SLA; mm^2^/g), specific root length (SRL; cm/g), and root tissue density (RTD; g/cm^3^). We selected five intact, fully extended, disease-free leaves without petioles from each seedling. Each sampled leaf was scanned (300 dpi resolution, 24 bit RGB) within 20 h after sampling, and the leaf area (LA; mm^2^) was measured using the Wanshen Leaf Processing System (version 2021; www.wseen.com, accessed on 23 July 2022) [57]. The complete root from each seedling was gently rinsed with water to remove the remaining soil before being spread as flat as possible in the root plate using tweezers and scanned (300 dpi resolution, 24-bit RGB). The root length (cm), surface area (cm^2^), and root volume (cm^3^) were measured using the Wanshen Root Processing System (version 2021). Each sampled leaf and root were weighed with an analytical balance (±0.001 g) to obtain leaf fresh mass, then dried at 80 °C for at least 48 h (until the difference between two consecutive measurements was less than 0.002 g) and weighed again for leaf dry mass and root dry mass. The SLA of each sampled leaf was calculated as the ratio of leaf area to the dry mass of the leaf. The SRL of each root was calculated as the ratio of root length to root dry mass, and the RTD of each root was calculated as the ratio of root dry mass to root volume. 

The total biomass (TB, g), including aboveground biomass (AB, g) (i.e., total dry mass of stem and leaf) and underground biomass (UB, g) (i.e., root dry mass) of each seedling, was weighed after drying at 80 °C for at least 48 h until the mass was unchanged. 

### 4.6. Data Analysis

We calculated the growth rate (*GR*) and the mortality rate (*M*) of seedlings under different treatments on islands or in greenhouses. The growth rate (*GR*) was calculated as follows:

*GR* = (*BA*_t1_ − *BA*_t0_)/*t* (1)

In (1), *t* represents the growth time between the first census and the last census during the P addition experiment, *BA*_t0_ represents the base area of seedlings at the first census, and *BA*_t1_ represents the base area of seedlings at the last census.

The mortality rate (*M*) was calculated using the changes in the number of individuals between the first census (*N*_t0_) and the last census (*N*_t1_) as follows:

*M* = 1 − *N*_t1_/*N*_t0_ (2)

We used the Analysis of Covariance (ANCOVA) method to test for significance in *GR* or *M* between the control treatment and the P-addition treatment while controlling for the influence of the island area (covariate). 

To investigate the effect of P addition on seedling growth and mortality when compared with the control treatment on different-sized islands, the mean value of the difference in *GR* or *M* between the P-addition treatment and the control treatment was calculated on each island for each species. This value was referred to as the relative growth rate or relative mortality rate. To further examine whether the island area could affect the relative growth rate or relative mortality rate after P addition, we applied a linear model, a quadratic linear model, and a logarithmic model to fit the relationship between the island area and the relative growth rate or relative mortality rate. The lowest value of Akaike’s information criterion corrected for small sample sizes (AICc) was used to select the best model [58], and only the best model was plotted. For AM tree *C. camphora*, since all seedlings (including control and P-addition treatment) on three islands died in the last census, we did not include the three islands in the analysis when testing the relationship between island area and relative growth rate. 

To test the differences in functional traits of seedlings with and without P addition, we used Tukey’s honestly significant difference (HSD) pair-wise post hoc test to test the significant differences in functional traits (SLA, SRL, and RTD) and biomass (TB, AB, and UB) between the P-addition treatment and the control treatment for each species in the greenhouse experiment. 

All statistical analyses were performed in R version 4.2.0 (www.r-project.org, accessed on 20 June 2023).

## 5. Conclusions

We observed that P addition (representing anthropogenic deposition) could increase the mortality of *C. camphora* (i.e., AM tree species) and *C. sclerophylla* (i.e., EcM tree species) seedlings on islands in an anthropogenically created subtropical archipelago. The relative growth rate of *C. camphora* seedlings under P addition was lower on smaller islands than on larger islands, suggesting a potential interaction between habitat patch size and P addition. We did not observe this relationship in *C. sclerophylla* seedlings. Using a separate greenhouse experiment, we explored possible mechanisms of P addition on seedling growth and mortality. We found that P addition could reduce both the specific root density of seedlings and the aboveground biomass of seedlings, indicating that P addition may affect the root tissue density of seedlings, thereby affecting their survival and growth. Our study reveals the synergistic effect of habitat fragmentation and P deposition, which may affect the regeneration of forest communities and biodiversity maintenance in fragmented habitats. 

## Data Availability

The raw data is available on request to the corresponding author.
